# Art of the Interwoven

**DOI:** 10.3201/eid3106.AC3106

**Published:** 2025-06

**Authors:** Byron Breedlove

**Affiliations:** Centers for Disease Control and Prevention, Atlanta, Georgia, USA

**Keywords:** Sarah Hunter, Jar-shaped Basket, basketry, basket weaving, Panamint Shoshone Tribe, Death Valley, vector-borne infections, hantavirus, plague

**Figure:**
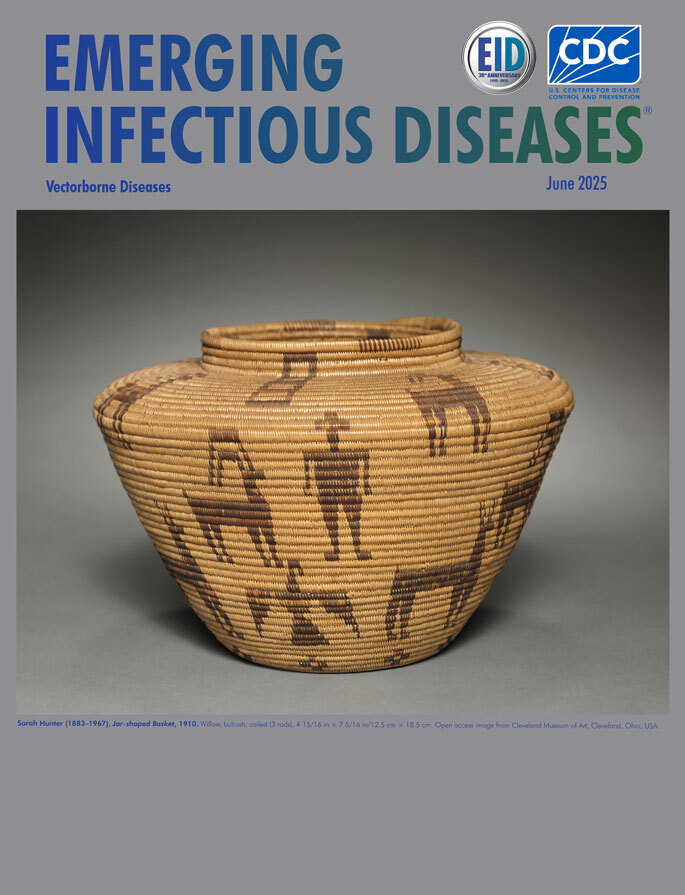
**Sarah Hunter (1883–1967), Jar-shaped Basket, 1910.** Willow, bulrush; coiled (3 rods), 4 15/16 in x 7 5/16 in/12.5 cm x 18.5 cm. Open access image from Cleveland Museum of Art, Cleveland, Ohio, USA.

In an article from the Gilcrease Museum website, anthropologist Jason Baird Jackson notes, “From the enormous diversity of American Indian peoples, speaking thousands of different languages and possessing distinct cultural traditions, inhabiting a great variety of natural settings, flowed a remarkable number of different basketry forms and techniques.” The Panamint Shoshone Tribe, today identified as the Timbisha Shoshone Tribe, “is historically known for some of the finest, tightly-coiled Native basketry on the continent,” according to the Portland Art Museum.

Appearing on this month’s cover is a jar-shaped basket created by Sara Hunter, who belonged to the Panamint Shoshone Tribe and was, according to the Cleveland Museum of Art, the last American Indian basket weaver to live in the Saline Valley on the edge of what is now Death Valley National Park in California. Eva Slater, an art historian and artist, notes in her book *Panamint Shoshone Basketry*, that “Sarah Hunter lived most of her life in the isolation of Hunter Canyon, the largest and most ancient of three camps in the valley.”

The Panamint Shoshones, a small branch of the Shoshone, have inhabited the Death Valley area for perhaps a thousand years and for centuries before Europeans ventured into the area. The US National Park service notes, “They hunted and followed seasonal migrations for harvesting of pinyon pine nuts and mesquite beans with their families. To them, the land provided everything they needed and many areas were, and are, considered to be sacred places.”

Initially, the Panamint created baskets for utilitarian purposes such as transporting and storing food, water, and possessions or for trapping birds and small game. Later, as settlers, silver miners, and others moved into the region, the Portland Art Museum explains that “the fine quality of Panamint baskets created a collectors’ market, which lasted well into the mid-twentieth century. For Panamint and other American Indian weavers, adapting utilitarian baskets to saleable ones preserved Native basket-making techniques and provided much needed income for many families still trying to live a traditional life.”

Slater writes that in the 1930s, Hunter “became known for her large oval vessels with zoomorphic design elements scattered playfully over the basket surface in petroglyph fashion revealing a relationship to Maggie Juaquin of Darwin, who was her sister.” Hunter, along with other basket makers in that region, would have gathered and prepared plants such as bulrush, bunchgrass, devil’s claw sumac, willow, and yucca for making baskets. The Cleveland Museum of Art, where this basket may be viewed, notes that “If stages of the process are not done properly and at the right time, color will be uneven and stitches will twist and split.”

The Cleveland Museum of Art recounts that “Hunter’s basketry is noted for geometricized motifs reminiscent of the animals depicted in petroglyphs on canyon walls in the Death Valley region. Here they include pronghorn mountain sheep, deer, and birds, along with humans, all created in light-brown bulrush against a honey-colored willow ground.” Animals adapted to living in this harsh ecosystem, like the plants found there, would have been essential resources for the Panamint tribe.

Living in proximity with nature also potentially increases human exposure to pathogens that could cause infection. In Death Valley, for example, zoonotic pathogens such as hantaviruses carried by rodents pose health threats to humans. The harsh, arid climate of this region confers, for now, protection from some vectorborne illnesses. The first recorded human-to-human transmission of plague in the United States was in San Francisco, California, in 1900. After fleas infected sylvatic rodents with plague, the disease spread across California, eventually reaching the Death Valley region. Western blacklegged ticks (*Ixodes pacificus*) that can transmit Lyme disease have been found in this region. Environmental changes and human encroachment may increase the prevalence of vectorborne diseases in Death Valley and similar areas.

Perhaps this jar-shaped basket showing the interconnection of animals, humans, and the environment in Death Valley and, created from weaving together various plants found in that harsh environment, may serve as a metaphor for One Health. One Health, as explained by the One Health High-Level Expert Panel, is globally recognized as “an integrated, unifying approach that aims to sustainably balance and optimize the health of people, animals, and ecosystems. It recognizes the health of humans, domestic and wild animals, plants, and the wider environment (including ecosystems) are closely linked and interdependent.” One Health is often described by words such as “integrated,” “intertwined,” and “interwoven.” From her work as basket maker and her knowledge of the land and its resources, Hunter would have intrinsically understood that underlying unity.

## References

[1] Burns JE, Metzger ME, Messenger S, Fritz CL, Vilcins IE, Enge B, et al. Novel focus of Sin Nombre virus in *Peromyscus eremicus* mice, Death Valley National Park, California, USA. Emerg Infect Dis. 2018;24:1112–5. 10.3201/eid2406.180089 29774841 PMC6004862

[2] California Department of Public Health. California compendium of plague control [cited 2025 May 12]. https://www.cdph.ca.gov/Programs/CID/DCDC/CDPH%20Document%20Library/CAPlagueCompendium.pdf

[3] California Department of Public Health. Lyme disease in California [cited 2025 May 5]. https://storymaps.arcgis.com/stories/f64d0c19a3ab42cf90e8ce38397e96e0

[4] Centers for Disease Control and Prevention. One Health basics [cited 2023 Oct 23]. https://www.cdc.gov/onehealth/basics/index.html

[5] Cleveland Museum of Art. Jar-shaped basket [cited 2023 Oct 2] https://www.clevelandart.org/art/1917.454

[6] Jackson JB. Article: Containers of tradition: Southeastern Indian basketry. Gilcrease Museum [cited 2023 Oct 19]. https://collections.gilcrease.org/articles/article-containers-tradition-southeastern-indian-basketry

[7] National Park Service. Death Valley. Land of a thousand stories: Native Americans [cited 2023 Oct 25] https://www.nps.gov/deva/learn/historyculture/people.htm

[8] Adisasmito WB, Almuhairi S, Behravesh CB, Bilivogui P, Bukachi SA, Casas N, et al.; One Health High-Level Expert Panel (OHHLEP). One Health: A new definition for a sustainable and healthy future. PLoS Pathog. 2022;18:e1010537. 10.1371/journal.ppat.1010537 35737670 PMC9223325

[9] Portland Art Museum. Panamint Coiled Baskets [cited 2023 Oct 19]. http://portlandartmuseum.us/mwebcgi/mweb.exe?request=record;id=261496;type=801

[10] Slater E. Panamint Shoshone Basketry. Morongo Valley (CA): Sagebrush Press; 2000. p. 15−16, 19−20, 55−61.

